# HLA Allele Frequencies and Association with Severity of COVID-19 Infection in Northern Italian Patients

**DOI:** 10.3390/cells11111792

**Published:** 2022-05-30

**Authors:** Franca Rosa Guerini, Elisabetta Bolognesi, Agata Lax, Luca Nicola Cesare Bianchi, Antonio Caronni, Milena Zanzottera, Cristina Agliardi, Maria Paola Albergoni, Paolo Innocente Banfi, Jorge Navarro, Mario Clerici

**Affiliations:** 1 IRCCS Fondazione Don Carlo Gnocchi ONLUS, Via Capecelatro, 66, 20148 Milan, Italy; fguerini@dongnocchi.it (F.R.G.); alax@dongnocchi.it (A.L.); lubianchi@dongnocchi.it (L.N.C.B.); antonio.caronni@gmail.com (A.C.); mzanzottera@dongnocchi.it (M.Z.); cagliardi@dongnocchi.it (C.A.); pabanfi@dongnocchi.it (P.I.B.); jnavarro@dongnocchi.it (J.N.); mario.clerici@unimi.it (M.C.); 2 UOC Immunotrasfusionale Azienda Ospedale, University of Padova, 35128 Padova, Italy; mariapaola.albergoni@aopd.veneto.it; 3 Department of Pathophysiology and Transplantation, University of Milan, 20122 Milan, Italy

**Keywords:** HLA, COVID-19, SARS-CoV-2, disease severity

## Abstract

HLA allelic distribution was analysed in a cohort of 96 Northern Italian subjects (53M/43F) (mean age 59.9 ± 13.3 years) from Lombardy who developed COVID-19 during the first two pandemic waves to investigate possible correlations between HLA molecules and disease severity. An important role of HLA- B and HLA-C loci in modulating the clinical severity of COVID-19 disease was identified. In particular, the HLA-B07 supertype was observed to be associated with a significant risk for severe disease; conversely, the HLA-B27 supertype and C*12:02 allele played a protective role as they were associated with milder disease. These associations were confirmed after applying a multinomial regression analysis to adjust the correlation for age, gender and comorbidities with COVID-19 severity. Though the power of results is limited by the small sample size, data herein contribute to shedding light on the role played by genetic background in COVID-19 infection.

## 1. Introduction

Coronavirus disease (COVID-19), a disease caused by severe acute respiratory syndrome coronavirus 2 (SARS-CoV-2), broke out in Wuhan (China) in December 2019 and very rapidly spread all over the world. In Italy, Lombardy (Northwest Italy), was the first region significantly impacted by the initial wave of COVID-19, with 75% of the total Italian cases of COVID-19 registered by 24th February 2020 [1]. The course of the disease is characterised by a heterogeneous spectrum of symptoms (from mild to moderate to severe to death) [2], which can differ greatly among individuals, thus suggesting a differential host response to infection. [3,4]. Different factors, including age, gender, smoking, immune status, the presence of chronic ailments and, more importantly, genetic background, might determine the severity of clinical manifestations and the outcome of SARS-CoV-2 infection [5,6,7,8,9,10,11]. In COVID-19, as is the case in any type of disease, a number of genetically determined immunological parameters determine the capability of the host to respond to infection. In particular, cellular immune responses are mediated by the ability of human leukocyte antigens (HLA) to display antigens that derive from the processing of pathogens on cells surfaces and present them to the appropriate T lymphocytes [12,13]. Different HLA molecules bind antigens with different affinities and present such antigens to T lymphocytes with different efficacies. As a consequence, HLA molecules directly influence the potency and the amplitude of immune responses and play a pivotal role in modulating susceptibility to infections and disease progression [14]. The role of the different HLA molecules in determining the severity of COVID-19 is still largely debated [15,16], and results are needed to clarify this topic. We analysed classical HLA class I-A/-B/-C and class II-DRB1/-DQB1 allelic distribution in relationship with severity of COVID-19 disease in a cohort of Northern Italian patients from Lombardy. Results, although stemming from a relatively small number of patients, show the presence of a clear correlation between HLA molecules and the clinical severity of COVID-19 infection.

## 2. Materials and Methods

### 2.1. Patients

A total of 96 Italian subjects (53M/43F) (mean age 59.9 ± 13.3) living in Lombardy and who developed COVID-19 during the first two pandemic waves were enrolled in the study after having recovered from SARS-CoV-2 infection. The study was run by the Fondazione Don Carlo Gnocchi Onlus (Milan, IRCCS Santa Maria Nascente, and Rovato, Centro Spalenza).

Individuals were clustered on the basis of COVID-19 severity classification [2] into three groups: (1) mild (*n* = 36): asymptomatic or flu-like symptoms without pneumonia manifestation; (2) moderate (*n* = 20): pneumonia manifestation in imaging without respiratory distress; and (3) severe (*n* = 40): pneumonia with respiratory distress treated with respiratory support (from continuous positive airway pressure to oro-tracheal intubation or tracheostomy). Demographic data and comorbidity conditions known to play a role in determining the clinical course of COVID-19 are described in results section. The study was approved by the local ethics committee (Comitato Etico, IRCCS Fondazione Don Gnocchi, Milano, protocol no. 03/20-05-2020), and patients or guardians provided written informed consent.

### 2.2. HLA—Class I and II Genotyping

Genomic DNA was isolated by phenol-chloroform extraction using standard procedures. HLA class I-A/-B/-C, and class II-DRB1/-DQB1 alleles typing was performed using HISTO TYPE SSP TYPING kits (BAG Diagnostic GMBH, Lich, Germany) according to the manufacturer’s instructions. Allele detection was performed after amplification in a VeritiPro^TM^ Thermal Cycler (Applied Biosystem by Thermo Fisher Scientific Inc., Waltham, MA, USA) by gel electrophoresis on 2% agarose gel. HLA-A and HLA-B alleles were also grouped into supertypes on the basis of their affinity for binding to the anchor peptide derived from viral antigen processing. Supertypes represent sets of molecules that share largely overlapping anchor peptide binding specificity [17].

### 2.3. Statistical Analysis

Association analysis of severity of disease course with age was performed by ANOVA, whereas contingency tables and chi-square analyses were applied to gender and comorbidity association evaluation. Allelic association analysis was performed to evaluate HLA genetic distribution between groups. N × 3 contingency tables where adopted, where: *n* = number of alleles detected for each HLA locus and 3 represents the clustered groups of patients (mild, moderate and severe). Chi-square analysis and *p*-value calculation were performed, Bonferroni correction for degree of freedom (df) was applied, df were calculated as “(*n*−1 alleles) × (*n*−1 groups)”, and pc values <0.05 were considered statistically significant. When an NX3 contingency table comparison result was statistically skewed, post hoc analysis were conducted using 2 × 2 tables to evaluate how single alleles were associated with disease severity, calculating *p* value with Fisher exact test (p_f_) and the odds ratio with a varying 95% interval of confidence (95% CI).

Finally, a multinomial regression model was applied to evaluate HLA allele association with disease severity course, considering COVID-19 severity (mild, moderate, severe) as the dependent variable, HLA alleles as factors and age, gender and comorbidities as covariates.

## 3. Results

### 3.1. HLA Allelic Distribution in Patients with Different COVID-19 Severity

Both demographic and comorbidity data of the enrolled persons were analysed in relationship with disease severity; results show that, as expected, moderate and severe disease was associated with older age (*p* < 0.001). Gender, hypertension, obstructive sleep apnea syndrome (OSAS) and diabetes mellitus were statistically associated with a more severe disease course as well (Table 1).

### 3.2. HLA Distribution Profiles of COVID-19 Patients

*HLA* class I-A/-B/-C -and class II-DQB1/-DRB1 genotype distribution was analysed next in relation to disease severity [2].

Results show that HLA-A allele distribution was not statistically different between the three analysed group of patients (Table S1) (pc = 0.24 df = 40). HLA-A supertype distribution did not reveal any statistical discrepancy in distribution between groups either (pc = 0.19 df = 10).

In contrast with these data, HLA-B allele distribution was significantly different between the three groups of patients, even after correction for 80 degrees of freedom (pc = 0.02) (Table 2). Post hoc comparison evidenced that this difference was principally due to the HLA-B*51:01 allele, which was carried by 4.4% of patients with mild disease severity, by 7.9% with moderate disease severity and by 15% of those with severe disease. Thus, statistically, a significant skewing in the frequency of the HLA-B*51:01 allele was detected when patients with severe COVID-19 disease were compared to those with mild disease (p_f_ = 0.04, pc = 1.40, df = 35 with an OR: 4.0 with a varying 95% CI: 1.2–18.50), though statistical significance was lost after Bonferroni’s correction.

Analysis of the HLA-B supertype distribution showed a statistical skewing between the three groups of patients as well (pc = 0.001, df = 12) (Table 3). This was mainly due to a higher frequency of the HLA-B07 supertype (which includes the HLA-B*51:01 allele), which was more frequently carried by patients with severe disease (46.3%) compared to those with mild disease (20.8%) (p_f_ = 0.002, pc = 0.01; df = 6, OR: 3.2, 95% CI:1.6–6.8). The HLA-B07 supertype was also more frequent in individuals with moderately severe COVID-19 infection (40.0%), (*p* = 0.05, pc = 0.30, OR: 2.5, 95% CI: 1.1–5.9) although this difference did not reach statistical significance after Bonferroni’s correction.

Conversely, the supertype HLA-B27 was less frequently carried by patients with moderate disease (2.5%) compared to those with mild disease (27.8%), (p_f_ = 0.001, pc = 0.006; OR: 0.06, 95% CI:0–0.4). The HLA-B27 supertype was also less frequent in individuals with severe COVID-19 infection (13.8%), (p_f_ = 0.05, OR: 0.4, 95% CI: 0.2–0.9), though statistical significance was not confirmed after correction for the degree of freedom (pc = 0.30)

HLA-C distribution was significantly different between the three groups of patients as well (pc = 0.002, df = 40) (Table 4). Specifically, the HLA-C*12:02 allele was more frequently carried by patients with mild disease (22.2%) than by those with moderate disease (2.5%) (p_f_ = 0.006, pc = 0.10, df = 17, OR: 0, 95% CI: 0.0–0.5), and was totally absent in those with severe COVID-19 infection (p_f_ < 0.001, pc < 0.001, df = 20, OR: 0, 95% CI: 0–0.15).

Finally, HLA-DRB1 and HLA-DQB1 allelic distribution was comparable in the three groups of patients (Tables S2 and S3).

### 3.3. Multinomial Regression Analysis of HLA Association Accounted for Comorbidities

Finally, as age and gender, as well as hypertension, OSAS and diabetes mellitus, were confirmed as risk factors for the likelihood of developing more severe COVID-19 infection in our case study (Table 1), a multinomial regression analysis was applied to the results to evaluate the possible HLA associations with disease severity, taking into account these risk factors. COVID-19 severity was imputed as a dependent variable, HLA alleles as factors, and age, gender and comorbidities as covariates. A significant association with disease severity was confirmed for the HLA-B07 (*p* = 0.02, Z = 2.36) and -B27 (*p* = 0.01, Z = −2.6) supertypes and the HLA-C*12:02 (*p* < 0.001, Z = −1.3 × 10^−6^) allele when patients with mild or severe disease were compared. Finally, the comparison between patients with moderate or mild disease confirmed that HLA-B27 was differentially carried by these two groups of individuals (*p* = 0.001, Z = −3.2).

## 4. Discussion

To date, different studies have suggested that HLA diversity plays a role in the severity of COVID-19 disease, but results are conflicting [15,16]. The genetic heterogeneity of the sample populations analysed may be one of the causes of the lack of clarity in this scenario. Moreover, COVID-19 severity was suggested to be modulated by a number of different geographical variables as well, including air pollutants [18] and ethnics and socio-economic factors [19]. To reduce genetic and geographic biases, we decided to focus our analyses on a group of ninety-six Italian individuals living in Lombardy who developed COVID-19 during the first two pandemic waves. Lombardy was the first region in Italy to be hit by the SARS-CoV2 pandemic and also the one that paid the highest price during the pandemic, in spite of the excellent quality of its medical and health facilities.

Results of our analyses suggest that HLA-B and HLA-C loci play an important role in modulating the clinical severity of COVID-19 disease. In particular, a possible protective role was observed to be mediated by the HLA-B27 supertype and by the HLA-C*12:02 allele. All these alleles were more frequently carried by patients who developed a mild form of the disease.

Conversely the HLA-B07 supertype could be considered a genetic risk factor for severe COVID-19. Moreover, the associations between these HLA alleles and disease severity were confirmed when age, gender and comorbidities risk factors were also considered as possible biases. It is noteworthy to observe that all the HLA alleles associated with severity of infection showed a gradient of frequency from mild to moderate to severe disease, further confirming their likely impact on determining the clinical outcome of COVID-19 disease.

The protective role of HLA-C*12:02 in SARS-CoV-2 infection is in agreement with a recent report by Detsika MG et al. [20] describing a lower frequency of hospitalised COVID-19 patients carrying the HLA-C*12 allele in a cohort of Greek origin. A putative protective role of HLA-C*12:02 allele was imputed to a high binding affinity with SARS-CoV-2 epitopes, confirming a better host response to the virus [14,21]. Conversely, a putative risk role for HLA-B07 is supported by the evidence of a lower binding affinity of this HLA molecule to viral antigens, leading to a higher probability of SARS-CoV-2 epitopes escaping CD8^+^ T cell immunosurveillance in HLA-B07 individuals [22]. Notably, HLA-B*51:01 is one of the alleles belonging to the HLA-B07 supertype and was also shown to be more frequently carried by patients with severe COVID-19 [23].

However, neither the risk nor the protective role suggested for other HLA alleles was confirmed by our results. The heterogeneity of the COVID-19-infected cohort of patients that were analysed in these studies is the most likely explanation for these discrepancies [15,16,24]. Moreover, it was suggested that different HLA alleles’ divergence in their ability to confer immunity could be the consequence of past infections with common coronaviruses, and that such an effect could depend on the time and type of the coronavirus responsible for past outbreaks [25]. Finally, it is important to note that the HLA region is known for its linkage disequilibrium; therefore, other genes very near to HLA could eventually be responsible for the association with COVID-19 severity.

An immediate point of criticism of our results is the small sample size, giving a low power of statistical analysis, which suggests a cautious interpretation of allelic association. The results herein, nevertheless, stem from analyses that include multiple variables that can modulate the outcome of infection and focalise on a group of individuals with a homogeneous geographical background. For this reason, these data may be a useful contribution in clarifying the role played by HLA heterogeneity in determining the severity of SARS-CoV-2 infection. Moreover, these results will be useful within global databases (e.g., http://www.hlacovid19.org/, accessed on April 15, 2022) for performing collaborative analyses that will lead to more conclusive results, avoiding the biases derived from the different allelic and haplotypic population frequencies.

## Figures and Tables

**Table 1:** Demographic data and comorbidity of 96 Italian patients clustered on the basis of COVID-19 severity classification in line with Chen and colleagues [2].

Disease	Mild (36)		Moderate (20)		Severe (40)		Total (96)		*p* Value
Mean Age (ds)	50.1	(9.6)	65.7	(15.4)	65.8	(9.62)	59.9	(13.3)	<0.001
Gender Male *n*%	14	38.9%	11	55.0%	28	70.0%	53	55.2%	0.025
Hypertension *n*%	5	13.9%	11	55.0%	28	70.0%	44	45.8%	<0.001
Active Smoking *n*%	7	19.4%	3	15.0%	12	30.0%	22	22.9%	0.35
Obstructive Sleep Apnea Syndrome (OSAS) *n*%	1	2.8%	2	10.0%	13	32.5%	16	16.7%	0.002
Diabetes Mellitus *n*%	0	0.0%	4	20.0%	8	20.0%	12	12.5%	0.02
Ischemic Heart Disease *n*%	2	8.3%	5	25.0%	6	15.0%	13	13.5%	0.12
Chronic Obstructive Pulmonary Disease *n*%	0	0.0%	3	15.0%	4	10.0%	7	7.3%	0.08
Malignancy *n*%	1	2.8%	2	10.0%	5	12.5%	8	8.3%	0.29
Asthma *n*%	4	11.1%	1	5.0%	1	2.5%	6	6.2%	0.29

*n*: number of patients affected by the disease before the diagnosis of COVID-19. ANOVA *p*-value analysis was adopted for age association with disease severity, whereas 3 × 2 contingency table was applied to calculate *p* value of association of gender and each comorbidity with disease severity.

**Table 2:** Frequencies of HLA-B alleles and HLA-B supertypes in 96 Italian patients clustered on the basis of COVID-19 severity classification in line with Chen and colleagues [2].

Supertype	Locus B	Mild (72)		Moderate (40)		Severe (80)		Total (192)	
		*n*	%	*n*	%	*n*	%	*n*	%
B07	*07:02*	3	4.4	5	13.2	3	3.8	11	5.9
B07	*07:05*	0	0.0	0	0.0	1	1.3	1	0.5
UND	*07:10*	0	0.0	0	0.0	1	1.3	1	0.5
B08	*08:01*	3	4.4	5	13.2	2	2.5	10	5.4
B62	*13:02*	4	5.9	3	7.9	3	3.8	10	5.4
B27	*14:02*	5	7.4	0	0.0	5	6.3	10	5.4
B62	*15:01*	0	0.0	1	2.6	2	2.5	3	1.6
B27	*15:03*	1	1.5	0	0.0	0	0.0	1	0.5
B62	*15:04*	0	0.0	0	0.0	1	1.3	1	0.5
B58	*15:17*	0	0.0	0	0.0	1	1.3	1	0.5
B62	*15:39*	1	1.5	0	0.0	0	0.0	1	0.5
B44	*18:01*	12	17.6	4	10.5	7	8.8	23	12.4
UND	*18:18*	0	0.0	1	2.6	0	0.0	1	0.5
B27	*27:01*	2	2.9	0	0.0	0	0.0	2	1.1
B27	*27:02*	1	1.5	0	0.0	0	0.0	1	0.5
B27	*27:03*	1	1.5	0	0.0	0	0.0	1	0.5
B07	*35:01*	4	5.9	2	5.3	13	16.3	19	10.2
B07	*35:02*	3	4.4	2	5.3	2	2.5	7	3.8
B07	*35:03*	0	0.0	1	2.6	1	1.3	2	1.1
B07	*35:08*	0	0.0	2	5.3	0	0.0	2	1.1
B07	*35:09*	0	0.0	0	0.0	1	1.3	1	0.5
B44	*37:01*	1	1.5	1	2.6	0	0.0	2	1.1
B27	*38:01*	5	7.4	1	2.6	3	3.8	9	4.8
B27	*39:01*	5	7.4	0	0.0	2	2.5	7	3.8
B44	*40:01*	2	2.9	0	0.0	1	1.3	3	1.6
B44	*40:02*	0	0.0	0	0.0	2	2.5	2	1.1
B44	*41:01*	0	0.0	2	5.3	0	0.0	2	1.1
B44	*44:02*	4	5.9	2	5.3	5	6.3	11	5.9
B44	*44:03*	2	2.9	1	2.6	0	0.0	3	1.6
B44	*44:05*	0	0.0	0	0.0	3	3.8	3	1.6
B44	*45:01*	1	1.5	0	0.0	0	0.0	1	0.5
UND	*47:01*	0	0.0	1	2.6	0	0.0	1	0.5
B44	*49:01*	2	2.9	0	0.0	1	1.3	3	1.6
B44	*50:01*	1	1.5	0	0.0	0	0.0	1	0.5
B07	*51:01*	3 *	4.4	3	7.9	12 *	15.0	18	9.7
B62	*52:01*	4	5.9	1	2.6	0	0.0	5	2.7
B07	*53:01*	1	1.5	1	2.6	2	2.5	4	2.2
B07	*55:01*	1	1.5	0	0.0	2	2.5	3	1.6
B58	*57:02*	0	0.0	1	2.6	0	0.0	1	0.5
B58	*58:01*	0	0.0	0	0.0	3	3.8	3	1.6
B27	*73:01*	0	0.0	0	0.0	1	1.3	1	0.5
								pc = 0.02, df = 80	

*n*: absolute number of alleles, %: allele frequency, UND: undefined supertype. *: severe vs. mild comparison: Fisher exact test: p_f_ = 0.04, pc = 1.40 after Bonferroni’s correction for df: = 35, OR: 4.0, 95% CI: 1.15–18.48.

**Table 3:** Comparison of HLA-B supertype frequencies of 96 Italian patients clustered on the basis of COVID-19 severity classification in line with Chen and colleagues [2].

Locus B	Mild (72)		Moderate (40)		Severe (80)		Total (192)				
Supertype	*n*	%	*n*	%	*n*	%	*n*	%	*p* Value	OR	95% CI
B07	*° 15	20.8	°16	40.0	* 37	46.3	68	15	* p_f_ = 0.002 * p = 0.01, df = 6 ° p_f_ = 0.05 pc = 0.30, df = 6	* 3.2 ° 2.5	* 1.6–6.8 ° 1.1–5.9
B08	3	4.2	5	12.5	2	2.5	10	3	ns		
B27	*° 20	27.8	° 1	2.5	* 11	13.8	32	20	* p_f_ = 0.05 * pc = 0.30, df = 6 ° p_f_ = 0.001 pc = 0.006, df = 6	* 0.4 ° 0.06	* 0.2–0.9 ° 0–0.4
B44	25	34.7	10	25.0	19	23.8	54	25	ns		
B58	0	0.0	1	2.5	4	5.0	5	0	ns		
B62	5	6.9	2	5.0	3	3.8	10	5	ns		
UND	4	5.6	5	12.5	4	5.0	13	4	ns		
									pc = 0.001, df = 12		

*n*: absolute number of alleles, %: allele frequency, UND: undefined supertype, *: severe vs. mild; °: moderate vs. mild comparison, p_f_: uncorrected Fisher exact test *p* value, pc: *p* value after Bonferroni’s correction for degree of freedom, df: degree of freedom, ns: not significant, OR: odds ratio, 95% CI: interval of confidence.

**Table 4:** Comparison of HLA-C frequencies of 96 Italian patients clustered on the basis of COVID-19 severity classification in line with Chen and colleagues [2].

Locus C	Mild (72)		Moderate (40)		Severe (80)		Total (192)				
	*n*	%	*n*	%	*n*	%	*n*	%	*p* Value	OR	95% CI
01:02	3	4.2	1	2.5	7	8.8	11	5.7	ns		
02:02	2	2.8	2	5.0	5	6.3	9	4.7	ns		
02:10	1	1.4	0	0.0	0	0.0	1	0.5	ns		
03:02	0	0.0	1	2.5	2	2.5	3	1.6	ns		
03.03	0	0.0	0	0.0	1	1.3	1	0.5	ns		
03:04	3	4.2	0	0.0	1	1.3	4	2.1	ns		
04:01	10	13.9	8	20.0	19	23.8	37	19.3	ns		
05:01	6	8.3	3	7.5	6	7.5	15	7.8	ns		
06:02	7	9.7	4	10.0	3	3.8	14	7.3	ns		
07:01	8	11.1	6	15.0	8	10.0	22	11.5	ns		
07:02	4	5.6	7	17.5	5	6.3	16	8.3	ns		
07:18	0	0.0	1	2.5	1	1.3	2	1.0	ns		
08:01	3	4.2	0	0.0	2	2.5	5	2.6	ns		
08:02	0	0.0	0	0.0	4	5.0	4	2.1	ns		
12:02	*° 16	22.2	° 1	2.5	* 0	0.0	17	8.9	* p_f_ < 0.001 * pc < 0.001, df = 19 ° p_f_ = 0.006 ° pc = 0.10, df = 17	* 0 ° 0	* 0–0.15 ° 0–0.5
12:03	2	2.8	2	5.0	7	8.8	11	5.7	ns		
14:02	1	1.4	1	2.5	1	1.3	3	1.6	ns		
15:02	2	2.8	0	0.0	5	6.3	7	3.6	ns		
15:05	0	0.0	0	0.0	2	2.5	2	1.0	ns		
16:01	4	5.6	1	2.5	1	1.3	6	3.1	ns		
17:01	0	0.0	2	5.0	0	0	2	1.0	ns		
									pc = 0.002, df = 40		

*n*: absolute number of alleles, %: allele frequency, *: severe vs. mild comparison, °: moderate vs. mild comparison, p_f_: Fisher exact test uncorrected *p* value, pc: *p* value after Bonferroni’s correction for degree of freedom, df: degree of freedom, ns: not significant, OR: odds ratio, 95% CI: interval of confidence.

## Data Availability

The raw data, will be available at 10.5281/zenodo.6563167.

## Supplementary Materials

The following supporting information can be downloaded at: https://www.mdpi.com/article/10.3390/cells11111792/s1. Table S1: frequencies of HLA-A alleles in 96 Italian patients clustered on the basis of COVID-19 severity classification established by Chen and colleagues [2]; Table S2: comparison of HLA-DRB1 frequencies in 96 Italian patients clustered on the basis of COVID-19 severity classification in line with Chen and colleagues [2]; Table S3: comparison of HLA-DQB1 frequencies of 96 Italian patients clustered on the basis of COVID-19 severity classification in line with Chen and colleagues [2].
