# Supplementary material for: HLA Allele Frequencies and Association with Severity of COVID-19 Infection in Northern Italian Patients
Source: Cells. 2022 May 30;11(11):1792. doi: 10.3390/cells11111792 (PMC9179900; doi:10.3390/cells11111792)
Supplement: Supplementary file 1 [file cells-11-01792-s001.zip › cells-1715096-supplementary.pdf]

Table S1: Frequencies of HLA-A alleles in 96 Italian patients clustered on the basis of COVID-19 severity classification in line with Chen and colleagues [2].

Supertype	Locus A	Mild (72)		Moderate (40)		Severe (80)		Total (192)	
		N	%	N	%	N	%	N	%
**A01**	*01:01*	8	11.1	5	12.5	5	6.3	18	9.4
**A01**	*01:03*	0	0.0	0	0.0	1	1.3	1	0.5
**A02**	*02:01*	18	25.0	6	15.0	19	23.8	43	22.4
**A03**	*03:01*	13	18.1	5	12.5	8	10.0	26	13.5
**A03**	*11:01*	2	2.8	1	2.5	9	11.3	12	6.3
**A24**	*23:01*	2	2.8	0	0.0	2	2.5	4	2.1
**A24**	*24:02*	5	6.9	7	17.5	12	15.0	24	12.5
**A01**	*25:01*	2	2.8	0	0.0	2	2.5	4	2.1
**A01**	*26:01*	3	4.2	3	7.5	1	1.3	7	3.6
**A01 A24**	*29:01*	3	4.2	1	2.5	2	2.5	6	3.1
**A01 A24**	*29:02*	0	0.0	1	2.5	1	1.3	2	1.0
**A01 A03**	*30:01*	7	9.7	1	2.5	1	1.3	9	4.7
**A01**	*30:02*	0	0.0	2	5.0	2	2.5	4	2.1
**A01**	*30:04*	0	0.0	0	0.0	1	1.3	1	0.5
**A03**	*31:01*	2	2.8	1	2.5	2	2.5	5	2.6
**A01**	*32:01*	4	5.6	4	10.0	3	3.8	11	5.7
**A03**	*33:01*	1	1.4	0	0.0	3	3.8	4	2.1
**A03**	*34:01*	0	0.0	0	0.0	1	1.3	1	0.5
**A03**	*68:01*	2	2.8	1	2.5	1	1.3	4	2.1
**A02**	*68:02*	0	0.0	1	2.5	4	5.0	5	2.6
**A02**	*69:01*	0	0.0	1	2.5	0	0.0	1	0.5
								pc=0.24 df= 40	

N: absolute number of alleles, %: allele frequency; pc: p value after Bonferroni's correction for degree of freedom, df: degree of freedom. HLA A supertype distribution: pc=0.19 df=10.

Table S2: Comparison of HLA -DRB1 frequencies in 96 Italian patients clustered on the basis of COVID-19 severity classification in line with Chen and colleagues [2].

Locus DRB1	Mild (72)		Moderate (40)		Severe (80)		Total (192)	
	N	%	N	%	N	%	N	%
01:01	10	13.9	3	7.5	8	10.0	21	10.9
01:02	0	0.0	0	0.0	3	3.8	3	1.6
03:01	8	11.1	5	12.5	8	10.0	21	10.9
04:01	1	1.4	0	0.0	0	0.0	1	3.1
04:05	5	6.9	0	0.0	1	1.3	6	0.5
07:01	0	0.0	0	0.0	1	1.3	1	10.9
08:01	10	13.9	6	15.0	5	6.3	21	1.6
08:03	2	2.8	0	0.0	0	0.0	2	1.0
08:04	1	1.4	0	0.0	2	2.5	3	1.6
09:01	0	0.0	0	0.0	1	1.3	1	1.6
10:01	1	1.4	0	0.0	2	2.5	3	15.1
11:01	0	0.0	1	2.5	2	2.5	3	9.4
11:02	13	18.1	6	15.0	10	12.5	29	0.5
11:03	2	2.8	0	0.0	0	0.0	2	1.0
11:04	1	1.4	0	0.0	0	0.0	1	0.5
12:01	3	4.2	4	10.0	11	13.8	18	1.6
13:01	0	0.0	0	0.0	1	1.3	1	1.0
13:02	4	5.6	3	7.5	5	6.3	12	1.0
13:03	0	0.0	1	2.5	7	8.8	8	6.8
14:01	2	2.8	0	0.0	1	1.3	3	2.1
14:54	2	2.8	0	0.0	0	0.0	2	5.2
15:01	0	0.0	1	2.5	1	1.3	2	0.5
15:02	3	4.2	6	15.0	4	5.0	13	6.8
16:01	3	4.2	0	0.0	1	1.3	4	2.1
16:02	1	1.4	3	7.5	6	7.5	10	5.2

pc=0.07 df=50

N: absolute number of alleles, %: allele frequency, pc: p value after Bonferroni's correction for degree of freedom, df: degree of freedom.

Table S3: Comparison of HLA -DQB1 frequencies of 96 Italian patients clustered on the basis of COVID-19 severity classification in line with Chen and colleagues [2].

Locus DQB1	Mild (72)		Moderate (40)		Severe (80)		Total (192)	
	N	%	N	%	N	%	N	%
02:01	8	11.1%	5	12.5%	8	10.0%	21	10.9%
02:02	10	13.9%	4	10.0%	3	3.8%	17	8.9%
03:01	26	36.1%	10	25.0%	25	31.3%	61	31.8%
03:02	0	0.0%	0	0.0%	2	2.5%	2	1.0%
03:03	1	1.4%	2	5.0%	4	5.0%	7	3.6%
04:02	4	5.6%	0	0.0%	2	2.5%	6	3.1%
05:01	11	15.3%	4	10.0%	14	17.5%	29	15.1%
05:02	1	1.4%	4	10.0%	6	7.5%	11	5.7%
05:03	1	1.4%	1	2.5%	1	1.3%	3	1.6%
06:01	3	4.2%	0	0.0%	0	0.0%	3	1.6%
06:02	3	4.2%	5	12.5%	3	3.8%	11	5.7%
06:03	4	5.6%	3	7.5%	6	7.5%	13	6.8%
06:04	0	0.0%	1	2.5%	5	6.3%	6	3.1%
06:09	0	0.0%	1	2.5%	1	1.3%	2	1.0%
								pc=0.14 df=26

N: absolute number of alleles, %: allele frequency, pc: p value after Bonferroni's correction for degree of freedom, df: degree of freedom.

Reference

Chen, T.; Wu, D.; Chen, H.; Yan, W.; Yang, D.; Chen, G.; Ma, K.; Xu, D.; Yu, H.; Wang, H.; et al. Clinical characteristics of 113 deceased patients with coronavirus disease 2019: Retrospective study. *BMJ* **2020**, *368*, m1091, https://doi.org/10.1136/bmj.m1091.
